# Supplementary material for: “It’s a slightly different vibe”. New pathways in condition-specific rehabilitation for people with new or existing joint pain
Source: PLoS One. 2025 Nov 13;20(11):e0336397. doi: 10.1371/journal.pone.0336397 (PMC12614521; doi:10.1371/journal.pone.0336397)
Supplement: S1 File — (DOCX) [file pone.0336397.s001.docx]

**Interview questions asked to participants regarding exercising to manage joint pain and participation in the Joint Pain Programme, in accordance with the theoretical domains framework (TDF) and COM-B model.**

| **Target Behaviour** | **COM-B component** | **TDF** | **Question** |
| --- | --- | --- | --- |
| **Exercise to manage joint pain** | **Capability - psychological** | Knowledge | What is your understanding of exercising to manage symptoms of joint pain? Did the 12-week programme improve your knowledge? How?  Prompt: Is there anything else you need to **know** to be able to sustain doing exercise now that you have completed the programme?  Was there any aspect of information that you felt was missing from the programme? |
|  |  | Memory, attention & decision making | To what extent (on a scale of 1-10) has exercising become routine? Has exercising always been routine for you? How will you ensure this routine continues moving forward?  Prompt – Going forward, is there anything that you need or that would help you to routinely exercise to manage your symptoms? |
|  |  | Behaviour Regulation | To what extent do you focus on making sure you exercise regularly, ignoring all temptations to do otherwise? Do you have personal strategies? How has the 12 week programme influenced this?  Prompt: Will you maintain this focus going forward? What would help? |
|  | **Capability - physical** | Skills | How important was it to you to have a rehab specialist dedicated to showing you how to exercise correctly? Was the face-to-face feedback helpful?  Prior to the programme, how confident were you about exercising in the gym/in a group class? On a scale of 1-10, how confident are you can continue to physically exercise to manage your symptoms of joint pain? Will lack of a dedicated Rehab Specialist influence this?  Prompt – Has the programme given you the necessary skills and confidence to be able to move forward and continuing exercising without the bespoke support of your rehabilitation specialist? What skills have you acquired? |
|  | | | |
|  | **Opportunity - social** | Social influences | Prompt: How does/would your family and friends support you in continuing to exercise to manage your joint pain?  How has the social aspects of the group influenced your participation? What does this look like moving forward? |
|  | **Opportunity - physical** | Environmental context & resources | Can you tell me about anything in your work or/and home environment that might influence whether you continue to exercise or not E.g. budget, time, location, weather  Are there any resources that you have used during the 12-week supervised programme that you will use in this next phase?  Will you continue to use NH after the subsidized period? If not, what will you do to continue to manage your joint pain? Can anything be done to support you after the subsidized period?  What are your thoughts about 24/7? Is this a helpful component of the programme?  What are your thoughts on the programme booklet that was issued to you? Will this be of any help moving forward? |
|  | | | |
|  | **Motivation - reflective** | Social, professional role & identify | Does exercising for health fit into your lifestyle and to your beliefs? To what extent would continuing to exercise to manage your joint pain be accepted by your friends and family?  Prompt: Do you think your family/friends influence how and when you exercise? What else would motivate you? |
|  |  | Beliefs about capability | Do you feel you are capable of continuing to exercise to manage your joint pain? Why/why not? What has made you capable?  Prompt: What are the barriers/facilitators to continuing to exercise to manage your joint pain? |
|  |  | Optimism | To what extent are you confident that any barriers you may have to exercising to manage your joint pain can be solved? |
|  |  | Beliefs about consequences | What do you think will happen if you continue to exercise to manage your joint pain?  Prompt: Discuss any benefits to exercising to reduce symptoms of joint pain? |
|  |  | Intentions | On a scale of 1-10, to what extent do you intend to continue exercising to manage your joint pain? What does the future look like for you in terms of those exercise intentions?  What will you do to ensure those intentions are carried out? Is there anything that would support you to carry out your intentions with respect to continuing to exercise? |
|  |  | Goals | Do you have any goals in relation to continuing to exercise?  Did you set goals in the 12 week programme? How did you find goal-setting, helpful or unhelpful? Difficult or easy? |
|  | **Motivation - automatic** | Reinforcement | Are there any incentives for you to continue to exercise to manage your joint pain? |
|  |  | Emotion | How will continuing to exercise to manage symptoms of joint pain would make you feel emotionally? |
|  |  |  | What will you miss most about the programme as you move into the next phase? |
|  |  |  | On a scale of 1-10, how motivated are you at this minute to continue exercising? |
|  | | | |
| **Reporting PROMs to Nuffield Health** | **Capability - psychological** | Knowledge | What is your understanding of the PROMS collected at 12/24 weeks following completion of the programme |
|  |  | Memory, attention & decision making | To what extent is giving feedback something you would normally do? |
|  | **Capability - physical** | Skills | To what extent are you confident that you can complete the PROMS at 12/24 weeks |
|  | | | |
|  | **Opportunity - physical** | Environmental context & resources | Is there anything that might help or hinder you continuing to report PROMS at 12/24 weeks E.g. budget, time, technology.  If NH wanted you to come back and report PROMs at 24 and 52 weeks, what could they do to help make this happen (thinking about people who might not continue to use the gym in the unsupported period) |
|  | | | |
|  | **Motivation - automatic** | Reinforcement | To what extent are there any incentives for you to report PROMS at 12/24 weeks. Do you think incentives would work? If yes, what incentives would work? |
