# Supplementary material for: “It’s a slightly different vibe”. New pathways in condition-specific rehabilitation for people with new or existing joint pain
Source: PLoS One. 2025 Nov 13;20(11):e0336397. doi: 10.1371/journal.pone.0336397 (PMC12614521; doi:10.1371/journal.pone.0336397)

Member Checking

Email sent to participants from researcher

Hello………..

We would once again like to thank you for participating in our Nuffield Health (in Partnership with Manchester Metropolitan University) project regarding your experiences regarding our joint pain programme. We would like to share with you the main findings (themes) of our project and would be very grateful if could kindly volunteer some feedback about this. Please note that to create a theme (project findings), we gathered the points that were made by most of the participants. **If you could offer comments as to whether you generally agree with the findings, or have any queries, your opinion would be very much appreciated. If you could do this by replying to this email (Jenny Alexanders – researcher).**


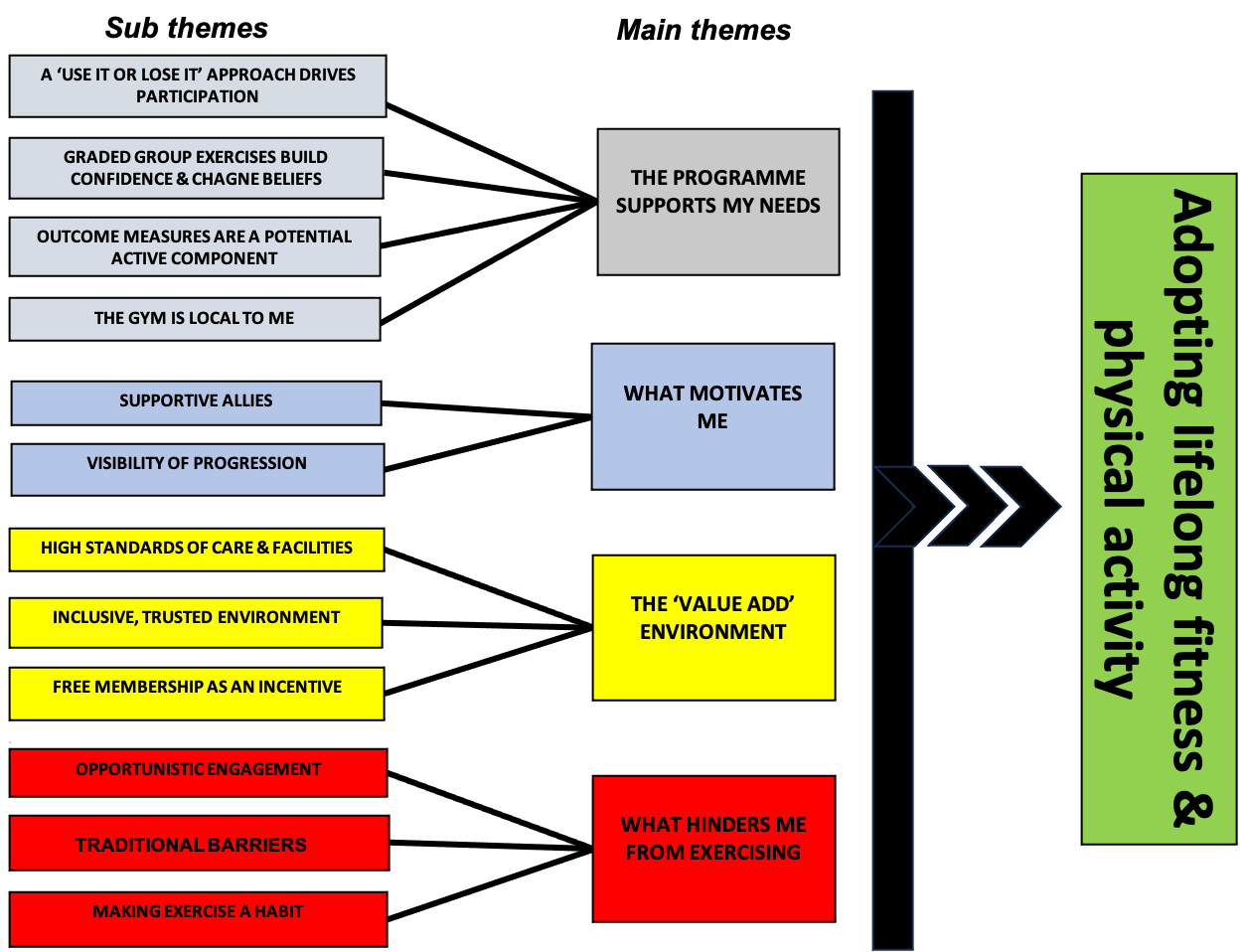


An explanation of the themes

**MAIN THEME – THE PROGRAMME SUPPORTS MY NEEDS**

This theme was to do with what people were saying about how the joint pain programme helps them. People spoke about the group exercise was motivating, the gym being local allowed them to get there with no problems, the outcomes (the results people felt e.g., becoming more mobile, feeling good, feeling stronger) empowered people.

**MAIN THEME – WHAT MOTIVATES ME**

This theme was to do with how exercising alongside other people with joint pain, stiffness etc helped motivate them and the way in which being able to do a little more (doing more exercise in the group sessions) motivated them.

**MAIN THEME – THE ‘VALUE ADD’ ENVIRONMENT**

This theme was generated because the participants spoke at length of the benefits Nuffield Health Gym brought. Benefits such as the free membership as part of the joint pain programme, high standards of facilities (clean, good working gym, pool etc) and a feeling inclusive (not feeling judged or embarrassed to work out at the Nuffield Health site.

**MAIN THEME – WHAT HINDERS FROM EXERCISING**

As well as themes which focused on the incentives, benefits and positives, it was important to balance this and report any barriers or aspects that could hinder people from adopting life long physical activity. The opportunistic engagement theme was the fact that people were finding out about the joint pain programme through word of mouth and social media but very little of them were referred through health care professionals – so this tells us that we could perhaps reach out to health care professionals to tell them about our programme. In addition, if healthcare professionals do not consider us when working with patients who have joint pain, then some patients may not join the programme. Some people reported that bad weather, family and work circumstances (titled traditional barriers) were often hinderances for them to partake in the programme. Other people spoke about the challenge making exercise a habit being a hinderance (basically it is hard to get going and making exercise a habit for life).


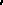

Supplement: S3 File — (DOCX) [file pone.0336397.s003.docx]
