# Supplementary material for: “It’s a slightly different vibe”. New pathways in condition-specific rehabilitation for people with new or existing joint pain
Source: PLoS One. 2025 Nov 13;20(11):e0336397. doi: 10.1371/journal.pone.0336397 (PMC12614521; doi:10.1371/journal.pone.0336397)
Supplement: Attachment — Submitted filename: Response to Reviewers.docx [file pone.0336397.s005.docx]

We would like to thank the editor and reviewers for their thoughtful and constructive feedback, which has been instrumental in improving the quality of this manuscript. We have carefully considered each of the comments and have made the corresponding revisions, which we believe has strengthened the clarity, depth, and overall impact of the paper. Please note that any signposting to the relevant sections of the manuscript is in reference to the tracked changed version. We have also made some minor formatting amendments such as displaying the ages as age ranges to better protect the anonymity of participants.

**Editors’ comments**

1. Please ensure that your manuscript meets PLOS ONE's style requirements.

**Authors’ Response:** Thank you for this comment. We believe the manuscript now meets these requirements.

2. In the online submission form, you indicated that your data will be submitted to a repository upon acceptance. We strongly recommend all authors deposit their data before acceptance, as the process can be lengthy and hold up publication timelines. Please note that, though access restrictions are acceptable now, your entire minimal dataset will need to be made freely accessible if your manuscript is accepted for publication. This policy applies to all data except where public deposition would breach compliance with the protocol approved by your research ethics board. If you are unable to adhere to our open data policy, please kindly revise your statement to explain your reasoning and we will seek the editor's input on an exemption.

**Authors’ Response:** Thank you for emphasising the importance of open data. We intend to upload the minimal dataset to a repository and plan to make the DOI publicly available upon acceptance of the manuscript. Please note, one participant did not consent to including their data in an open repository, therefore their data will not be included.

3. When completing the data availability statement of the submission form, you indicated that you will make your data available on acceptance. We strongly recommend all authors decide on a data sharing plan before acceptance, as the process can be lengthy and hold up publication timelines. Please note that, though access restrictions are acceptable now, your entire data will need to be made freely accessible if your manuscript is accepted for publication. This policy applies to all data except where public deposition would breach compliance with the protocol approved by your research ethics board. If you are unable to adhere to our open data policy, please kindly revise your statement to explain your reasoning and we will seek the editor's input on an exemption. Please be assured that, once you have provided your new statement, the assessment of your exemption will not hold up the peer review process.

**Authors’ Response:** Thank you for this comment. We believe this comment relates to comment 2 and as such, our response is similar. We intend to upload the minimal dataset to a repository and plan to make the DOI publicly available upon acceptance of the manuscript.

4. We note that this data set consists of interview transcripts. Can you please confirm that all participants gave consent for interview transcript to be published? If they DID provide consent for these transcripts to be published, please also confirm that the transcripts do not contain any potentially identifying information (or let us know if the participants consented to having their personal details published and made publicly available).

**Authors’ Response:** All but one participant provided consent for the anonymised dataset to be published. The data from this participant will not be included. We have reviewed the intended dataset for publication to ensure no identifiable information will be included.

**Reviewer 1 Comments**

Reviewer 1: The manuscript is well written and sets out a clear rationale for this study. Below are a few comments:

1. Authors should consider being specific about the study condition. Abstract background describes MSKs, but introduction describes osteoarthritis. MSK conditions encompass a wide range of Musculoskeletal conditions and thus will be a better background description for the study. Further, only four participants reported osteoarthritis as reason for referral.

**Authors’ Response:** Thank you for your comment. The introduction section of the manuscript has included more global reference to MSKs disorders which also included OA as being one of those named conditions.

2. Line 65 – Suggest using UK National Health Service in the first instance for global readers

**Authors’ Response**: Thank you for your comment. This has been changed.

3. Well written introduction, but authors do not provide any rationale for using the three behavioural theories. Why was the TDF used to inform interviews? Why was the TTM and SEM used to interpret findings? How does these behavioural theories add to the gap in the literature? What other behavioural theories have been used to explore this research area?

**Authors’ Response:** Thank you for your comment. In response, we have clarified the rationale for the selection and application of each behavioural model within the Introduction.

4. Line 124- Unclear what cross-sectional study is referred to here

**Authors’ Response:** Thank you for your comment. We have changed the description of this to give it more clarity.

5. Line 135-136 A clear definition for ‘have no uncontrolled medical conditions’ is required. One participant is reported having COPD. How do the authors justify that this is not an uncontrolled medical condition? How is an uncontrolled medical condition assessed?

**Authors’ Response**: Thank you for raising this important aspect. We have now included some additional details around how a controlled / uncontrolled medical condition is assessed on pages 8 and 9 (line 168-173).

6. Line 185 suggest ‘A use it or lose it principle’ rather than approach

**Authors’ Response**: Thank you for your comment. We agree that this term better captures the essence of the sub-theme. The change can be seen on page 12.

7. Line 185-196 Authors may consider adding this sub theme to Theme 2 given that participants describe being motivated by a concern of losing physical function

**Authors’ Response**: Thank you for this comment, we believe this improves the flow of the paper and consistency in the thematic definitions. We have actioned this suggestion accordingly. In line with this change, the quote related to motivation through social exchange has also been moved to Theme 2 (Page 16 line 376).

8. Results – suggest additional description for quotes to aid reader e.g. (P (number), gender MSK condition etc rather than P(number)

**Authors’ Response**: Thank you for this comment. We have now included additional descriptors alongside quotes.

9. Line 214 – Suggest ‘value of outcome assessments’ as title instead

**Authors’ Response**: Thank you for your comment. We agree the wording improves the clarity of the sub-theme. The change can be seen on page 13 and reflected in the Discussion also.

10. Line 252 – Period missing at the end of sentence

**Authors’ Response**: Thank you for highlighting this. A period has been added (page 15 line 305).

11. Discussion is too long winded. An approach may be to discuss the key findings (rather than each theme/ subtheme) within the context of the two frameworks. The SEM diagram is detailed enough to help reduce some of the text in this section. Authors should consider making reference to this diagram. The discussion should only highlight the key findings within the context to the literature.

**Authors’ Response**: Thank you for your comment. The discussion section has been substantially revised to enhance clarity and precision. Key points have been restructured and rearticulated to provide greater focus and coherence throughout.

12. Key recommendations should include recommending for the JPP intervention. The findings have explored some barriers to engagement with the intervention. Recommendations to therefore improve the attendance can be highlighted here

**Authors’ Response**: Thank you for this comment. We have now included recommendations specific to improving outcomes and attendance of the JPP intervention.

**Reviewer 2 Comments**

1. The title should be like this: ‘Condition-specific rehabilitation for people with new or existing joint pain’

**Authors’ Response**: Thank you for your feedback. We believe the structure, duration, and level of support provided by the JPP represent a novel pathway that warrants clear distinction. While we acknowledge the presence of similar programmes in some UK communities, these typically do not incorporate the same structured format, educational components, or digital signposting offered by the JPP. Moreover, the specific expertise and training of Rehabilitation Specialists (RSs) is integral to the programme's delivery and are unique to this organisational context.

2. Results should be the results, not only written on the theme. The inside of the theme will be presented as results or findings.

**Authors’ Response:** Thank you for your observation. In line with conventional qualitative reporting, we structured the results section thematically, illustrating each theme with participant quotes to substantiate and contextualise our interpretations. These themes were then critically explored in the discussion section, where we developed and contextualised them in relation to existing literature and theoretical frameworks.

3. The strength, weakness and limitation should be addressed properly.

**Authors’ Response**: Thank you for your comments. We have aimed to present the strengths and limitations of the study in an honest and balanced manner to ensure transparency and support the rigour of our reporting. We appreciate your feedback and have reviewed this section again to confirm that it accurately reflects the scope and context of the work.

4. Table 1: Age and year with conditions should descriptively address where mean and SD should be addressed.

**Authors’ Response:** Thank you for your comment. The mean and SD of participant age and year living with conditions has been added to the Note. of Table 1.

5. English grammar correction is important.

**Authors’ Response:** Thank you for your comment. The manuscript has been thoroughly proofread to improve grammatical accuracy and punctuation, ensuring clarity and consistency throughout the sections.
