# Supplementary material for: “It’s a slightly different vibe”. New pathways in condition-specific rehabilitation for people with new or existing joint pain
Source: PLoS One. 2025 Nov 13;20(11):e0336397. doi: 10.1371/journal.pone.0336397 (PMC12614521; doi:10.1371/journal.pone.0336397)
Supplement: Attachment — Submitted filename: Rebuttal Letter.docx [file pone.0336397.s006.docx]

We would like to thank the editor for reviewing the revised manuscript and providing further feedback. We have reviewed comments and have made corresponding revisions where required. Please note that any signposting to the relevant sections of the manuscript is in reference to the tracked changed version.

**Journal Requirements:**

1. If the reviewer comments include a recommendation to cite specific previously published works, please review and evaluate these publications to determine whether they are relevant and should be cited. There is no requirement to cite these works unless the editor has indicated otherwise.

*Authors’ Response:* Thank you for this comment. We believe we have only included relevant citations throughout the document. The reviewers did not provide any recommendations to cite specific work in their response.

2. Please review your reference list to ensure that it is complete and correct. If you have cited papers that have been retracted, please include the rationale for doing so in the manuscript text, or remove these references and replace them with relevant current references. Any changes to the reference list should be mentioned in the rebuttal letter that accompanies your revised manuscript. If you need to cite a retracted article, indicate the article’s retracted status in the References list and also include a citation and full reference for the retraction notice.

*Authors’ Response:* Thank you for emphasising the importance of justifying the use of retracted papers. We have reviewed the references we have included using both EndNote and manually on Retraction Watch and can confirm that none are retracted.

**Additional Editor Comments:**

1. Yes, now the manuscript can be accepted after the English correction and format correction are properly done.

*Authors’ Response:* Thank you for this comment. We have identified formatting errors such as incorrect use of quotation marks (p15 line 298; p25 line 534-535) and additional space between words (p25 line 519-520). We have also identified two formatting issues within the reference list which we have now addressed (p26 line 551 and 568). We have removed a duplicate reference which appeared as a new citation (p5 line 91 and p27 line 586-588) and have updated the corresponding numbers to all other references and in text citations which follow. We have also ensured all journals within the references align where possible with the NLM abbreviations. Lastly, we have made one change to the author affiliations.

2. While revising your submission, please upload your figure files to the Preflight Analysis and Conversion Engine (PACE) digital diagnostic tool, [https://pacev2.apexcovantage.com/](https://eur03.safelinks.protection.outlook.com/?url=https%3A%2F%2Ftrack.editorialmanager.com%2FCL0%2Fhttps%3A%252F%252Fpacev2.apexcovantage.com%252F%2F1%2F010f01984271a2ec-20400bea-1885-4663-a933-db3700b5f653-000000%2FxIt1EdgGXuraj7Iw_o2TuVMqEOIHDOL3yoBDk98HXVs%3D221&data=05%7C02%7Cpanayiotis.michael%40nuffieldhealth.com%7C2460e8272c854a19db2808ddcb996f6c%7C1badfb81fefa4bfc85d8eece023a15f5%7C0%7C0%7C638890582049683075%7CUnknown%7CTWFpbGZsb3d8eyJFbXB0eU1hcGkiOnRydWUsIlYiOiIwLjAuMDAwMCIsIlAiOiJXaW4zMiIsIkFOIjoiTWFpbCIsIldUIjoyfQ%3D%3D%7C0%7C%7C%7C&sdata=KJvWx%2Bc9t2mxza8AcJvjrnLc9UdpXQrb91g8hHzAl%2B8%3D&reserved=0). PACE helps ensure that figures meet PLOS requirements. To use PACE, you must first register as a user. Registration is free. Then, login and navigate to the UPLOAD tab, where you will find detailed instructions on how to use the tool. If you encounter any issues or have any questions when using PACE, please email PLOS at [figures@plos.org](mailto:figures@plos.org). Please note that Supporting Information files do not need this step.

*Authors’ Response:* Thank you for reminding us of the tool. We have uploaded our figures to generate the correct formatting requirements, with the new files being provided with the re-submission.
