# Supplementary material for: “It’s a slightly different vibe”. New pathways in condition-specific rehabilitation for people with new or existing joint pain
Source: PLoS One. 2025 Nov 13;20(11):e0336397. doi: 10.1371/journal.pone.0336397 (PMC12614521; doi:10.1371/journal.pone.0336397)
Supplement: Attachment — Submitted filename: Rebuttal_Letter_auresp_3.docx [file pone.0336397.s007.docx]

We would like to thank the editor for reviewing the revised manuscript and providing further feedback. We have reviewed comments and have made corresponding revisions where required. Please note that any signposting to the relevant sections of the manuscript is in reference to the tracked changed version.

**Journal Requirements:**

1. If the reviewer comments include a recommendation to cite specific previously published works, please review and evaluate these publications to determine whether they are relevant and should be cited. There is no requirement to cite these works unless the editor has indicated otherwise.

*Authors’ Response:* Thank you for this comment. We believe we have only included relevant citations throughout the document. The reviewers did not provide any recommendations to cite specific work in their response.

2. Please review your reference list to ensure that it is complete and correct. If you have cited papers that have been retracted, please include the rationale for doing so in the manuscript text, or remove these references and replace them with relevant current references. Any changes to the reference list should be mentioned in the rebuttal letter that accompanies your revised manuscript. If you need to cite a retracted article, indicate the article’s retracted status in the References list and also include a citation and full reference for the retraction notice.

*Authors’ Response:* Thank you for emphasising the importance of justifying the use of retracted papers. We have reviewed the references we have included using both EndNote and manually on Retraction Watch and can confirm that none are retracted. We have also ensured our reference list is complete and correct.

**Additional Editor Comments:**

1. Please submit the manuscript after thorough English correction within the next 15 days.

*Authors’ Response:* Thank you for this comment. We have carefully reviewed the manuscript for grammar, punctuation and clarity, and have made necessary English language corrections throughout the document:

Abstract (lines 25-27; 35-36)

Introduction (lines 51-52; 54; 56-57; 79-80; 92; 96; 100; 104)

Methods (lines 157; 162; 181-182; 191)

Results (lines 201-202; 241; 270; 276; 283-284; 302; 304-305; 329)

Discussion (lines 360; 375; 379; 392; 398; 421-422; 427; 438; 443; 446; 468; 503-504; 517)

Conclusion (lines 520-521)

Key Recommendations (lines 542-544)
